# Hunting and persecution drive mammal declines in Iran

**DOI:** 10.1038/s41598-022-22238-5

**Published:** 2022-10-22

**Authors:** Gholam Hosein Yusefi, José Carlos Brito, Mahmood Soofi, Kamran Safi

**Affiliations:** 1 CIBIO, Centro de Investigação em Biodiversidade e Recursos Genéticos, InBIO Laboratório Associado, Universidade do Porto, Campus de Vairão, 4485-661 Vairão, Portugal; 2 BIOPOLIS Program in Genomics, Biodiversity and Land Planning, CIBIO, Campus de Vairão, 4485-661 Vairão, Portugal; 3 Departamento de Biologia, Faculdade de Ciências, Universidade do Porto, 4099-002 Porto, Portugal; 4 Mohitban Society, No. 91, Moghaddas Ardebili Str., Tehran, 19859-14747 Iran; 5 Department of Conservation Biology, J. F. Blumenbach Institute of Zoology and Anthropology, University of Goettingen, Bürgerstr. 50, 37073 Göttingen, Germany; 6 CSIRO Land and Water, PMB 44, Winnellie, Darwin, NT 0822 Australia; 7 Department of Migration, Max Planck Institute of Animal Behavior, 78315 Radolfzell, Germany; 8 Department of Biology, University of Konstanz, 78467 Konstanz, Germany

**Keywords:** Ecology, Zoology

## Abstract

The negative impacts of human activities on biodiversity are well documented. However, extinction risk studies incorporating direct human threats particularly direct killing remain limited. Here, we evaluate the potential role that direct killing through hunting and persecution, indirect human threats via land-use change, and environmental and species traits such as reproductive rate and trophic level among others, may play in driving mammal species to extinction. Based on data for 156 mammal species from Iran, we applied generalized linear models to investigate correlates of extinction risk for: (1) all mammalian species, (2) large- and (3) small-bodied species. We show that hunting vulnerability is the most important predictor to affect extinction risk across all species. We also found that the small-bodied species are impacted by indirect human influence, whereas large species are highly affected by direct killing. Overall, the extrinsic environmental factors and intrinsic species traits had lower importance in our models. Our study gives insight into the dominant role of direct killing on mammal species decline and extinction, emphasizing the need to account for the different sources of threats when analysing the correlates of extinction risk.

## Introduction

Human activities (e.g., exploitation and land transformation) continue to impact biodiversity in many regions worldwide. In consequence, many species are threatened with extinction because of these activities causing the negative impacts on populations and habitats^1,2^. While hunting and poaching mainly for food or trophy as well as persecution in retaliation for human-wildlife conflicts or other purposes directly decimated the number of individuals and populations, the unabated and increasing expansion of agriculture and infrastructure developments (e.g., roads, buildings) further reduces available natural habitats for many species regionally and globally^3^. Despite the urgency and magnitude of the ongoing human driven extinction, the knowledge of how our activities in detail have led to the observed species population decline and extinction is limited with many gaps in knowledge. Especially important is the knowledge on how different sources of human threats interact with the various species’ traits to define their risk of extinction (the probability of extinction of a species)^4–6^. In particular, the role of direct killing (e.g., hunting and persecution) in the current species extinction crisis remain poorly quantified compared to the indirect human threats such as land-use change and the intrinsic as well as extrinsic species traits such as reproductive rate and trophic level among others^7,8^.

Within mammals, a review of 68 comparative studies of extinction risk reported that while descriptions of species’ biology, ecology and morphology as well as environmental and habitat variables are widely used in predictive modelling of risk of extinction, but the influence of human threats have rarely been considered or modelled simultaneously^9^. Those very few studies on extinction risk that did include human threats did so only considering indirect human threats such as human population density within the distributional ranges (e.g.,^10,11^). Direct threats such as hunting pressure and persecution have remained little explored (but see^12^). This is despite the fact that for effective conservation beyond and in addition of understanding the susceptibility associated with the species traits it is pivotal to uncover the full range of threats behind current populations and species declines^5,8^.

**Figure 1:**
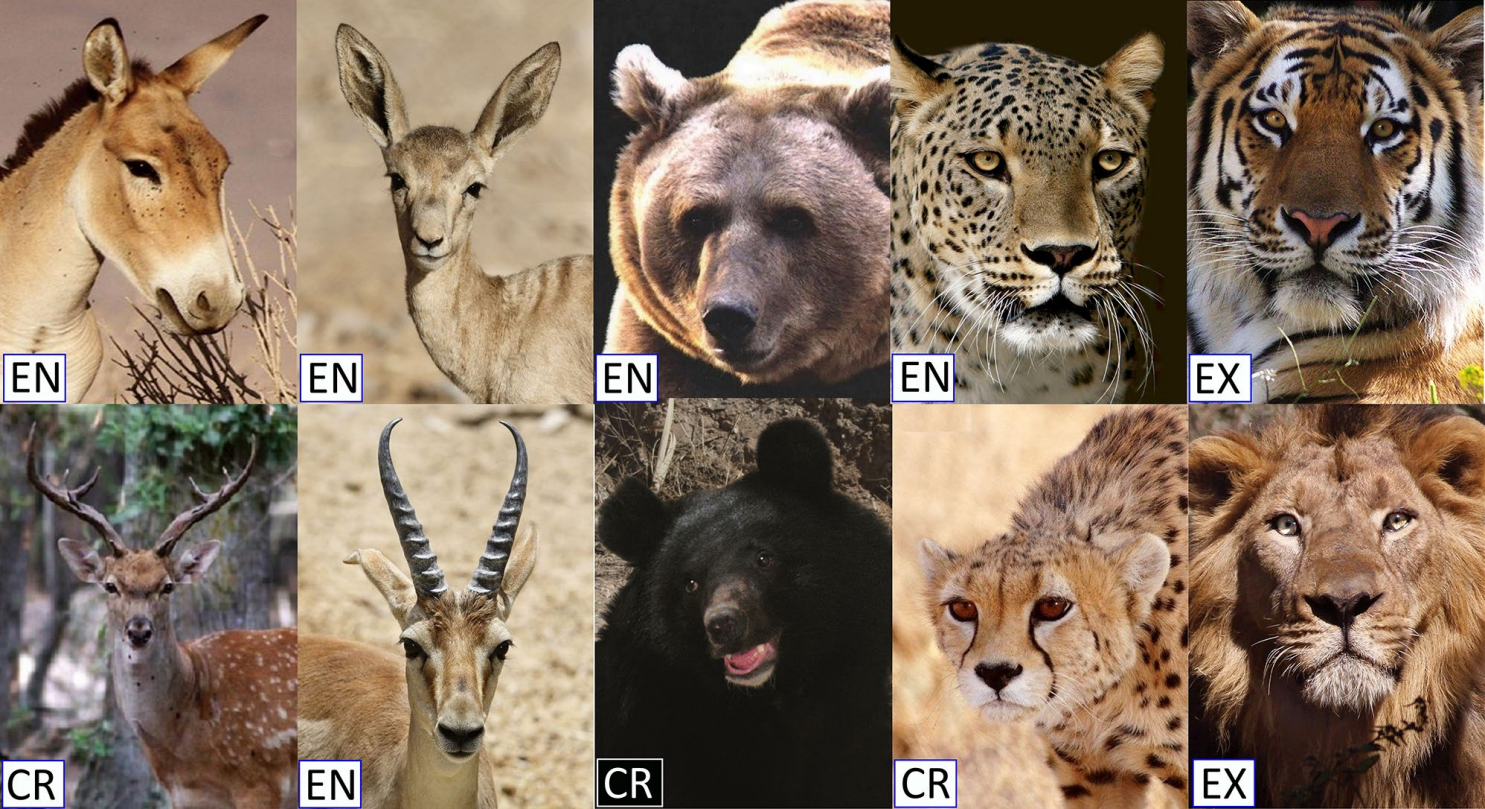
Photographic examples of threatened large mammals. Top row left to right: Onager or Persian wild ass (*Equus hemionus onager*), Goitered or Persian gazelle (*Gazella subgutturosa*), brown bear (*Ursus arctos*), Persian leopard (*Panthera pardus saxicolor*), and Caspian tiger (*Panthera tigris virgata*). Bottom row left to right: Persian fallow deer (*Dama mesopotamica*), Chinkara or Indian gazelle (*Gazella bennettii*), Asiatic black bear (*Ursus thibetauns thibetauns*), Asiatic cheetah (*Acynonix jubatus venaticus*), and Persian lion (*Panthera leo persica*). The national endangerment classification for each species (adapted from Yusefi et al. ^13^) noted on the image. Status categories are endangered (EN), critically endangered (CR) and extinct (EX). Photo credits: Fariborz Heidari.

The Iranian terrestrial mammal fauna offers an ideal setting to analyse the extinction patterns and to investigate the effects of increasing human activities on species decline and extinction for four reasons. First, the country is rich in term of mammal diversity as it is home to 192 terrestrial species from 34 families, perhaps one of the most diverse countries in the entire Palaearctic realm^13^. Second, Iran represents one of the last strongholds for large mammals in southwest Asia, where a substantial portion of regions’ large mammals’ distribution ranges with their remaining populations are currently confined to. Third, human pressure has increased substantially in the last five decades causing a population decline and local extinctions, resulting in around 28% of the 188 mammalian species being either threatened (25 species) or near threatened (26 species)^13^. Fourth, the country’s well-preserved large mammals (Fig. 1) provide the ideal backdrop for investigating the effects of direct killing on extinction risk. The wide range of still remaining species of carnivores and ungulates (39 species)^13^ form the basis for the high conservation interest that the mammalian fauna of Iran has for the wider region, which necessitates the understanding of factors affecting the extinction risk to inform conservation efforts. Little is known about the risks faced by mammals in the southwest Asia, wherefore the mammal community of Iran can serve as testing ground to understand the current condition of a diverse assemblage of mammals.

Here we characterize the relationship between direct (hunting/persecution) and indirect threats (human presence and land-use), intrinsic species traits (e.g., litter size) and extrinsic factors (e.g., habitat breadth) in Iranian terrestrial mammal’s extinction risk, using a regional assemblage of terrestrial mammals and an integrative approach incorporating multiple predictor variables (Table 1) as well as phylogenetic information. We explicitly stress the potential influences that direct killing might have on vulnerability to extinction. Modelling extinction risk at local scale is of interest because the correlates of extinction risk can vary across scales^14^ and because they can reveal patterns that would be masked in large-scale comparisons^9^. But more importantly, local scale studies are also of interest given that human impacts variables might be most important at this scale^15^. Finally, since practical conservation takes place at national scales^16^ the results from analyses at this scale produce more targeted outcomes which could have more influence on conservation practices^17^.

**Table 1:** Description of variables used in the extinction risk analyses in terrestrial mammals of Iran. See Supplementary Table S1 for details of variables.

Predictor type	Variable	Code	Definition *	Classes	Effect **
Morphology (intrinsic)	Adult body mass	ABM	Mass of adult (gr)	Continuous	Positive
Life-history (intrinsic)	Gestation length	GL	N days of non-inactive foetal growth	Continuous	Positive
	Litter size	LS	N offspring born per litter per female	Continuous	Negative
Ecology (extrinsic)	Diet breadth	DB	N dietary categories eaten by each species	Vertebrate, invertebrate, fruit, flowers/ nectar/pollen, leaves/branches/bark, seeds, grass and roots/tubers	Negative
“	Habitat breadth	HB	N habitat types used by each species	Above ground dwelling, aquatic, fossorial and ground dwelling	Negative
“	Trophic level	TL	Position that an organism occupies in a food chain, measured using any qualitative or quantitative dietary measure	1—Herbivore (not vertebrate and/or invertebrate); 2—Omnivore (vertebrate and/or invertebrate plus any of the other categories); 3—Carnivore (vertebrate and/or invertebrate only)	Positive
Environment (extrinsic)	Actual evapotranspiration	AET	Primary productivity index (mm)	Continuous, larger values indicate highest surface water evaporation	Negative
Anthropogenic (indirect)	Human influence Index	HII	Index combining human population pressure, land-use/infrastructure, and accessibility	Continuous, larger values indicate highest possible human influence	Positive
Anthropogenic (direct)	Hunting vulnerability measured via IUCN threats	HV	Measure of hunting pressure	1—Rarely/never hunted or persecuted (hunting is not a threat for species); 2—Occasionally hunted or persecuted (hunting is not main threat); 3—Often hunted or persecuted (hunting is main threat)	Positive
Response variable	National red list	IUCN	Conservation status	0—LC, Least Concern; 1—NT, Near Threatened; 2—VU, Vulnerable; 3—EN, Endangered; 4—CR, Critically Endangered; 5—RE, Regionally Extinct	NA

*See PanTHERIA database^46^ for an extended description of the variables and their sources.

**The known relationships between predictors and extinct risk.

NA, not applicable.

## Results

The variables most strongly associated with risk of extinction in all mammals, in rank of order, were hunting vulnerability (HV) (*β* = 0.64, 95% CI = 0.46 to 0.82, RI = 1.00), actual evapotranspiration (AET) (*β* = 0.18, 95% CI = 0.00 to 0.35, RI = 0.75), and litter size (LS) (*β* = − 0.42, 95% CI = − 0.70 to − 0.14, RI = 1.00). However, we found no significant effects of human influence index (HII) (*β* = 0.15, 95% CI = − 0.06 to 0.34), diet breadth (DB) (*β* = 0.14, 95% CI = − 0.10 to 0.39) and habitat breadth (HB) (*β* = − 0.08, 95% CI = − 0.42 to 0.27) on extinction risk (Table 2).

**Table 2:** Model averaging results for the best candidate models from QAICc-based (Quasi Akaike Information Criterion Corrected for small sample size) model selection (delta < 2) for large, small and all (pooled data) mammal species in Iran. Relative importance values represent the comparison of the contribution of each covariate in the original models and the simulated models. *β* is the estimated beta coefficient. Variable codes explained in Table 1.

Model	Parameter	*β* ± unconditional SE	Confidence interval	Exp (*β*)	*P*-value	Relative importance	
						Original	Simulation
**All mammals (n = 156)**							
	Intercept	− 0.93 ± 0.14	(− 1.21, − 0.65)	0.39	0.00	–	–
	AET	0.18 ± 0.09	(0.00, 0.35)	1.19	0.05	0.75	0.75
	DB	0.14 ± 0.12	(− 0.10, 0.39)	1.15	0.24	0.25	0.25
	HB	− 0.08 ± 0.18	(− 0.42, 0.27)	0.92	0.66	0.09	0.09
	HII	0.14 ± 0.10	(− 0.06, 0.34)	1.15	0.17	0.39	0.39
	HV	0.64 ± 0.09	(0.46, 0.82)	1.89	0.00	1.00	1.00
	LS	− 0.42 ± 0.14	(− 0.70, − 0.14)	0.65	0.00	1.00	1.00
**Large mammals (n = 39)**							
	Intercept	0.13 ± 0.18	(− 0.23, 0.975)	1.13	0.49	-	-
	ABM	0.33 ± 0.12	(0.09, 0.58)	1.39	0.01	1.00	1.00
	AET	0.25 ± 0.11	(0.02, 0.47)	1.28	0.03	0.75	0.75
	HB	− 0.43 ± 0.32	(− 1.08, 0.23)	0.65	0.20	0.28	0.28
	HV	0.41 ± 0.17	(0.06, 0.76)	1.50	0.02	0.87	0.87
	LS	− 0.39 ± 0.19	(− 0.77, − 0.01)	0.67	0.04	0.73	0.73
**Small mammals (n = 117)**							
	Intercept	− 1.41 ± 0.19	(− 1.80, − 1.02)	0.24	0.00	–	–
	ABM	0.09 ± 0.17	(− 1.79, − 1.02)	1.09	0.59	0.05	0.05
	AET	− 0.28 ± 0.20	(− 0.67, 0.12)	0.75	0.17	0.51	0.51
	DB	− 0.25 ± 0.21	(− 0.68, 0.17)	0.77	0.24	0.23	0.23
	GL	0.29 ± 0.17	(− 0.06, 0.63)	1.33	0.10	0.45	0.45
	HII	0.38 ± 0.13	(0.12, 0.64)	1.46	0.00	1.00	1.00
	HV	− 0.18 ± 0.22	(− 0.61, 0.25)	0.83	0.41	0.17	0.17
	LS	− 0.26 ± 0.19	(− 0.64, 0.13)	0.77	0.19	0.15	0.15

Predictors of extinction risk in large and small mammals showed different patterns. In large mammals, HV (*β* = 0.41, 95% CI = 0.06 to 0.76, RI = 0.87), adult body mass (ABM) (*β* = 0.33, 95% CI = 0.09 to 0.58, RI = 1), AET (*β* = 0.25, 95% CI = 0.02 to 0.47, RI = 1), and LS (*β* = − 0.39, 95% CI = − 0.77 to − 0.01, RI = 0.73) were identified as important predictors, in rank of order, of decline and extinction. By contrast, in small mammals’ model, we found only HII likely to significantly influence conservation status (*β* = 0.38, 95% CI = 0.12 to 0.64, RI = 1) (Table 2). Furthermore, our results from simulation exercises showed that the estimates and the RI of the parameters remained consistent compared to the original models results (Table 2). No phylogenetic signal was detected for none of the tested variables, indicating that a phylogenetic correction was unnecessary. The Moran’s *I* coefficients test revealed also no phylogenetic autocorrelation (Moran’s *I* test all: *p* > 0.05).

## Discussion

Extinction risk studies provide valuable information for conservation actions^18^, but to be effective, they must focus not only on species biological traits but also on the threats behind current species and populations declines^4,5^. Our study based on a comprehensive analysis of potential factors that might be associated with extinction risk of Iranian mammals indicates that direct threats from human through hunting and persecution is the most important predictor of decline and extinction in this local assemblage. This result adds to the limited available information for regional scales (but see^19,20^), and is consistent with recent studies (e.g.,^7^) that reveal hunting as an important predictor of decline for mammals’ species globally. Our results further show that the drivers of increased extinction risk in large- and small-bodied mammals are different. Such that large mammals are highly impacted by direct killing, but small mammals are mainly affected by indirect human threats such as habitat loss.

The direct process of overexploitation and the more indirect process of habitat loss respectively affected different species groups differently. Irrespective of the obvious differences in how conservation action has to mitigate overexploitation versus habitat loss^3^ and their innate and practical differences in the IUCN threat classifications schemes, generally, both of these anthropogenic threats are referred to as “direct” threats in the extinction risk studies^4^. Accordingly, in many studies (e.g.,^10,11,21–24^) proxies such as human population density, human footprint index and human influence index (that collectively combine the impacts of multiple factors of human presence, activity and disturbances) are treated as “direct” threat to species. These factors however actually tend to affect species indirectly through the reduction of natural habitats and prompting habitat loss, thus in reality capturing indirect impacts of human activities. (Still, the results of these studies show that these factors play a significant or key role in reducing the population of mammals and their extinction.) The inclusion of direct killing as a separate variable in our models showed that the potential effects of these two kinds of threats are indeed different, and each can be a key driver of decline and extinction in different sets of species. The different patterns found here for direct killing through hunting/persecution and human influence through presence/activity suggest that the assumption of the key role of human indirect threats in mammal decline and extinction might be too simplistic.

Our results show that most of the hunting pressure is on large mammals, as they are more likely to be targeted for hunting and persecution (i.e., more susceptible to mortality) compared to small mammals^25–27^. This is not surprising because body size is usually strongly linked with extinction risk in declining mammals and has been identified as one of the most consistent predictors of extinction vulnerability^9^. Indeed, previous analyses of the conservation status of mammals based on the IUCN Red List data has shown that overexploitation through poaching and/or hunting is highly associated with the global threat status of large mammals^8^ and with the extinction risk of 60% of the large terrestrial threatened mammals^7^. This pressure increases when considering that large mammals have low capacity to recover, as they usually display lower population densities and low reproductive rates (longer gestations or weaning ages or lesser litter sizes)^21^. Ripple et al.^7,25^ report that hunting leads to greater decline of threatened species in comparison to habitat loss, particularly in developing countries based on the effects of hunting on endangerment status of world terrestrial mammals. Another study by Benítez-López et al.^5^ shows that mammal abundance declines by over 80% in hunting areas compared to non-hunting areas. This emphasizes the need of urgent action to prevent direct killing of species both through controlling hunting and poaching, as well as finding ways to decrease persecution caused by human-wildlife conflicts, especially in countries where large mammal populations are highly threatened^26^.

We also found that litter size was negatively associated with extinction risk in both the total assemblage and the large-bodied species but not the small-sized ones. Litter size is known as an important indicator of life-history speed^22^, meaning that the time that species need to recover from a change (decline) is tightly related to this variable. The interactions found between litter size and human direct pressure suggest that the probability of extinction in our case was not only related to the threat itself, but also to the time that it takes to recover from such threat. These results are consistent with those found by Cardillo et al.^23^, in the sense that interactions of some species traits with threats tend to augment their extinction risk. The lower importance of litter size in small-sized species can be explained by the fact that the vast majority of small mammals (except bats) have large litters per year. While actual evapotranspiration (AET) interpreted as a proxy of primary or environmental productivity that are mean monthly AET values from across species ranges, has been shown to negatively correlate with extinction risk (e.g.,^28^), our results show the opposite where with increasing AET risk of extinction also increases, in both the total assemblage and the large-bodied species. This relationship probably suggests that the species occurring in high primary productivity areas (i.e. areas with high AET) represent remnant of threatened populations, restricted to high quality patches^28,29^. This is consistent with the expectation that species go extinct locally first in suboptimal habitats. But it might also indicate that the species adapted to high AET are particularly large-bodied and/or the high AET areas are the ones under particular threat of, for instance, land use change. Available data suggests that Iran (overall an arid to semi-arid country) is characterized with limited precipitation and suffer from decreasing primary productivity and thus the country seems to support generally lower population densities of larger species^30^, which might reflect the importance of the primary productivity in our study.

Unlike previous studies showing that small mammal extinctions are driven –at broader scales– by environmental features^31^ or –at coarser scales– by life history traits^20^, we found that indirect human impact is the key factor for driving extinction in this group. This finding is consistent with our hypothesis that increased risk of extinction in small mammals might be facilitated by human indirect threats, such as habitat destruction rather than direct hunting/persecution^32^. In our case, this pattern can be explained by the fact that many large mammals, despite having large distribution ranges, currently are forced to live in remote areas where human population pressure, land-use through cropland/pastureland and infrastructure expansions, as well as road/light intensifications (factors that collectively contribute to the HII variable) are low. This became evident when we compared the size and the location of the current distribution areas and the levels of indirect human impact to which small-bodied species are exposed in comparison to the large-bodied species. While many small species in Iran display small ranges (40,000 to 93,000 km^2^) and persist in areas heavily affected by human activities (HII: 23.4–32.6), many threatened large mammal species still display very large ranges (149,000–1,600,000 km^2^; Supplementary Table S1) despite the population declines experienced over the last decades and are currently restricted to areas with low human impacts (HII: 8.3–17.1). This is the case for several large and threatened species, including the Asiatic cheetah, Asiatic black bear (*Ursus thibetanus*), brown bear (*U. arctos*), gazelles (*Gazella subgutturosa* and *G. bennettii*), leopard, wild sheep (*Ovis vignei*) and wild goat (*Capra aegagrus*). Thus, among the smaller species those in proximity to humans are more prone to decline and suffer from a higher extinction risk through indirect human impact. At present, investigations of the consequences of anthropogenic pressure on mammals, particularly the comparative influence of the HII components, are lacking (similarly to what has been recently done by Hill et al.^33^), providing an important area in need of future research. The distinction in dispersal capacity between small- and large-bodied species in this respect, may also explain the higher impact of human activity that relate to habitat loss and degradation (indirect threats) on smaller mammals since they have limited dispersal abilities.

There are few caveats to note. The extent of direct killing by humans is difficult to measure^7^. This is especially true for the number of poached and persecuted animals, as poaching is an illegal phenomenon and hence it is notoriously hard to detect them, which usually are not officially documented, compared to the hunting information that is often available. Here, we measured vulnerability based on the global threat classifications (IUCN categories) and verified it by local information. Although this variable positively correlated with species decline and extinction, we are aware that the measure used here may not the best one to capture the intensity of hunting pressure and that the actual number of hunted, poached and persecuted animals should provide a better indication of hunting pressure. However, such data are not available in many areas in the world and more so for data-poor regions, such as Asia. Alternative measures, such as number of hunting permits (yearly issued by Iran Department of Environment) or number of rangers, have their own drawbacks. The former excludes poached and persecuted mammals, thus it does not quantify the real number of killed animals, and it is only available for game animals (ungulates), while the latter can be a biased measure, as hunting pressure will depend more on guard effectiveness in relation to their number.

Our study is the first one to systematically investigate the patterns and processes of extinction and threat in a large number of Iranian mammals. We found that species that often were reported to be persecuted or hunted are those which are most likely to become extinct in the near future. This is despite the fact that since the establishment of the DoE in the 1950s, unlawful hunting and persecution of wildlife being banned in Iran and the majority of the large mammal’s species being listed as “protected” and regulations implemented to prohibit their unauthorized hunting inside and outside protected areas^34^. In addition, the number of protected areas (and the size of the areas under protection) has been continuously increasing since the 1950s (currently 246 areas;^35^). Apparently, these conservation policy instruments have been ineffective to halt population declines observed in large mammals^35^, and to combat illegal hunting effective implementation of the measures and regulations are urgently needed.

The observed strong correlation between hunting vulnerability and threat status suggests that more effective protection should be assigned to large mammals. This is especially important because many areas containing large mammal assemblages merit conservation attention globally, as it has been shown that only a small portion (8%) of the land area that still retains complete assemblages of large mammals is well protected^36^. Indeed, several lines of evidence support the primacy of hunting and/or persecution as the main causes of population decline and extinction in the country, including two charismatic large cats, the Caspian tiger (*Panthera tigris virgata*) and the Asiatic lion (*Panthera leo persica*) that have gone extinct only in the past decades, and several other large mammals experienced range contractions and have become locally extinct^13,30^. Currently, hunting particularly affects ungulate species^37,38^, which in turn, may further affect predators/carnivores via food scarcity^28,29^, as in the case of Asiatic cheetah^39^. The warning status of ungulates in Iran reflects the concerns for large herbivores at the global level^40^. These species require immediate conservation efforts to reverse their continuous decline, as it has been shown that conservation action does prevent ungulate species from going extinct^41^.

The information on the relative importance of different threatening processes and factors responsible for the current decline or extinction is critical to aid conservation efforts, especially because most conservation actions are operating at national scales, where the outcomes may be directly translated into local conservation policies and practices^19^. Although the negative impacts of local hunting/poaching on a few large mammals have been documented previously (e.g.,^37,38^), we present for the first time a strong quantitative support for the devastating effects of this factor on decline and extinction on much of the country’s mammal diversity. Thus, our findings could be used to aid national conservation planning. Nevertheless, due to the scarcity of current knowledge regarding the number of hunted and persecuted animals together with an expected increasing trend of direct killing^7^, effects of hunting and persecution on mammals (and other taxa) should be the subject of deeper research and become a matter of management concern^4^. Until this shortfall is addressed, guidelines for wildlife conservation should consider more strict regulations to control direct killings in order to protect the last remaining species and populations. Furthermore, future conservation strategies must consider differences in the drivers of extinction between large and small mammals and the varying susceptibilities of different groups to different threatening processes. Likewise, conservation actions will be more effective if focused on mitigating particular threatening processes, rather than wide-ranging conservation managements. Finally, although our study focuses on the potential influences that direct killing might have on vulnerability to extinction among Iranian mammals, other threats such as climate change and invasive species may also play critical roles in species declines and endangerments that require special attention for conservation.

## Methods

### Study area

Iran covers an area of 1.65 million km^2^ and located in southwest Asia, between the latitudes of 25°30' and 40° north and the longitudes of 44° and 63°30' east. The elevation ranges from -28 to 5671 m and annual rainfall varies from < 100 mm in Central Basin up to > 2,000 mm in the Caspian Sea coasts (Iran Meteorological Organization, http://www.irimo.ir). The topography is very complex (> 50% above 1000 m) which makes Iran a heterogeneous country with a large variety of habitat types from the temperate humid forests to cold mountains and extreme deserts. The country is a proverbial bridge between the Mediterranean and Arabia on one side and Central Asia and Indian region on the other side. A major part of the land is covered by the central basin, which has a high physiographic complexity with several scattered large and small mountains. This area is mainly surrounded by the Alborz Mountains in the north and the Zagros Mountains in the west. Iran is one of the richest and most complex regions in western Asia from a biodiversity point of view, given that the North and West of the country categorised as two (Caucasus and Irano-Anatolian) of only seven Asia’s biodiversity hotspots^30,42^.

### Database construction

**Figure 2:**
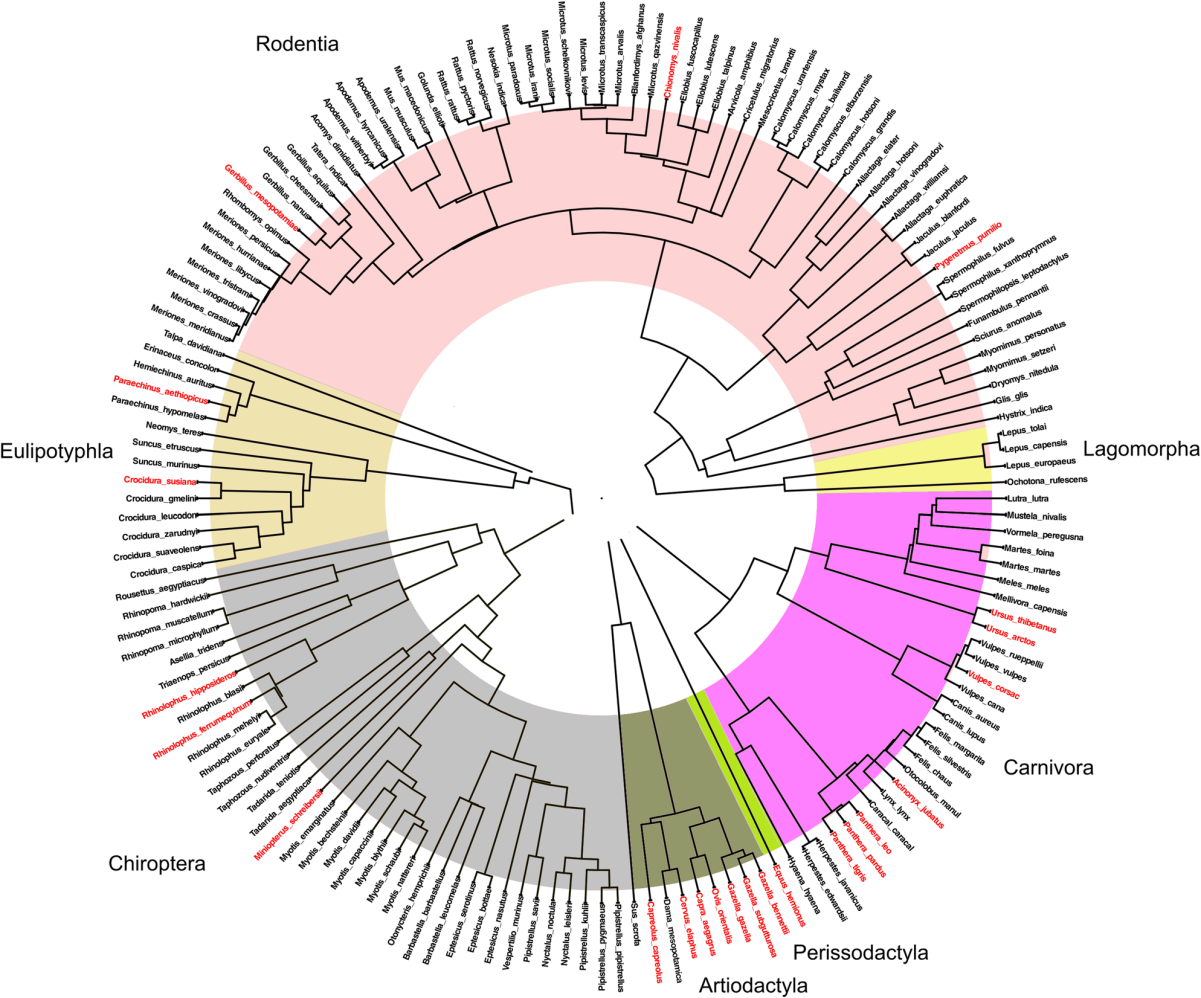
Phylogenetic tree of the 156 mammal species belonging to seven orders and 31 families used in the comparative analysis. The colour of the labels (scientific names) of the tree show threatened (red colour) and non-threatened (black colour) species. The phylogeny is built based on a modification of the species-level supertree of mammals generated by ^43^.

As a source for mammal phylogeny, we used an updated species-level supertree^43^ to derive the pairwise (cophenetic) phylogenetic distances using the R^44^ ‘ape’ package^45^. We were able to incorporate 179 species of Iranian mammals (from 192 species listed in^13^) into this supertree after editing species lists and reconciling synonym species in both data sets. We then built a phylogenetic tree for 156 species belonging to seven orders and 31 families (Fig. 2), after excluding species with unknown threat status (Data Deficient) (see below).

We used variables representative of the species’ morphology, life-histories, ecologies and environmental features from PanTHERIA database^46^ (Table 1). Among all of the available variables (*n* = 42), we selected those for which data were available for > 50% of the cases, in order to reduce bias in analyses that may arise from the missing data^47^. This dataset was supplemented with further data from the peer-reviewed literature. Further, in the case of Eulipotyphla, Chiroptera and Rodentia orders we used genus values or genus averages for missing species wherever the data was available for other species in the same genus. The resulting variables were adult body mass, diet breadth, gestation length, habitat breadth, litter size, trophic level, and actual evapotranspiration rate (see details in Supplementary Table S1), all are among important extinction risk predictors in mammals^9^. We divided these variables into two categories: intrinsic (morphological, life-history) and extrinsic (ecological, environmental). Here we distinguished diet breadth and habitat breadth traits that are associated with specialization of species from the life-history traits that are associated with reproductive speed such as litter size, gestation length, and weaning age.

As an index of the intensity of direct human impact, we used hunting vulnerability. We quantified hunting vulnerability using individual species accounts from the IUCN Red List of Threatened Species^3^ following the same approach as in previous studies (e.g.,^7,48^). We quantified the hunting vulnerability in three categories based on threat from hunting: (1) rarely/never hunted or persecuted (species that were rarely or never hunted in any of the above ways), (2) occasionally hunted or persecuted (if they were not preferred game species or actively persecuted species), and (3) often hunted or persecuted (if they were game species or actively persecuted species) (see details in Supplementary Table S1). IUCN list the threats believed to impacting the species and are classified according to the IUCN Red List authorities^3^. We further verified such information (vulnerability condition) against local sources (data). We searched the reliable reports on hunting and/or persecution of mammals in Iran using the Google search engine. We examined reports using combinations of “hunting”, “poaching”, “shooting animals”, “killing animals”, “persecution of animals”, ‘hunter”, “poacher”, and “poachers arrest” in national media news websites (in Persian). Additionally, we searched Iranian literature such as Firouz et al.^30^ to ensure that the species are appropriately classified as “hunting vulnerable” in the scale of the country.

The intensity of indirect human impact was considered via the HII^49^. HII combines the impacts of multiple factors of human presence and activity (e.g., population density, land-use, accessibility/roads) and is suitable to quantify the effects of human disturbances and indicates the proportion of species range that has undergone anthropogenic landcover transformation (the higher the HII, the more human impacts and the more local extinction)^49^. First, for each of the 179 species, we measured species’ range size as the extent of occurrence (EOO) (i.e., minimum convex polygon), based on the recent distributional review available containing over 14,000 mammalian species occurrences (Supplementary Fig. S1)^13^. These occurrences data were collected from published scientific literature (> 850), online databases, grey literature, unpublished data, field observations, plus distributional data of 43 medium and large species from more than 400 areas under protection (see details in^13^). EOO estimations were performed in ArcGIS ver. 10.6^50^. Then, the HII of Iran was extracted from the global dataset and overlaid with the EOO for each species (Supplementary Table S1 and Fig. S2). The species-specific mean HII experienced by each species was calculated using ‘raster’^51^, ‘rgdal’^52^, and ‘maptools’^53^ R packages.

**Figure 3:**
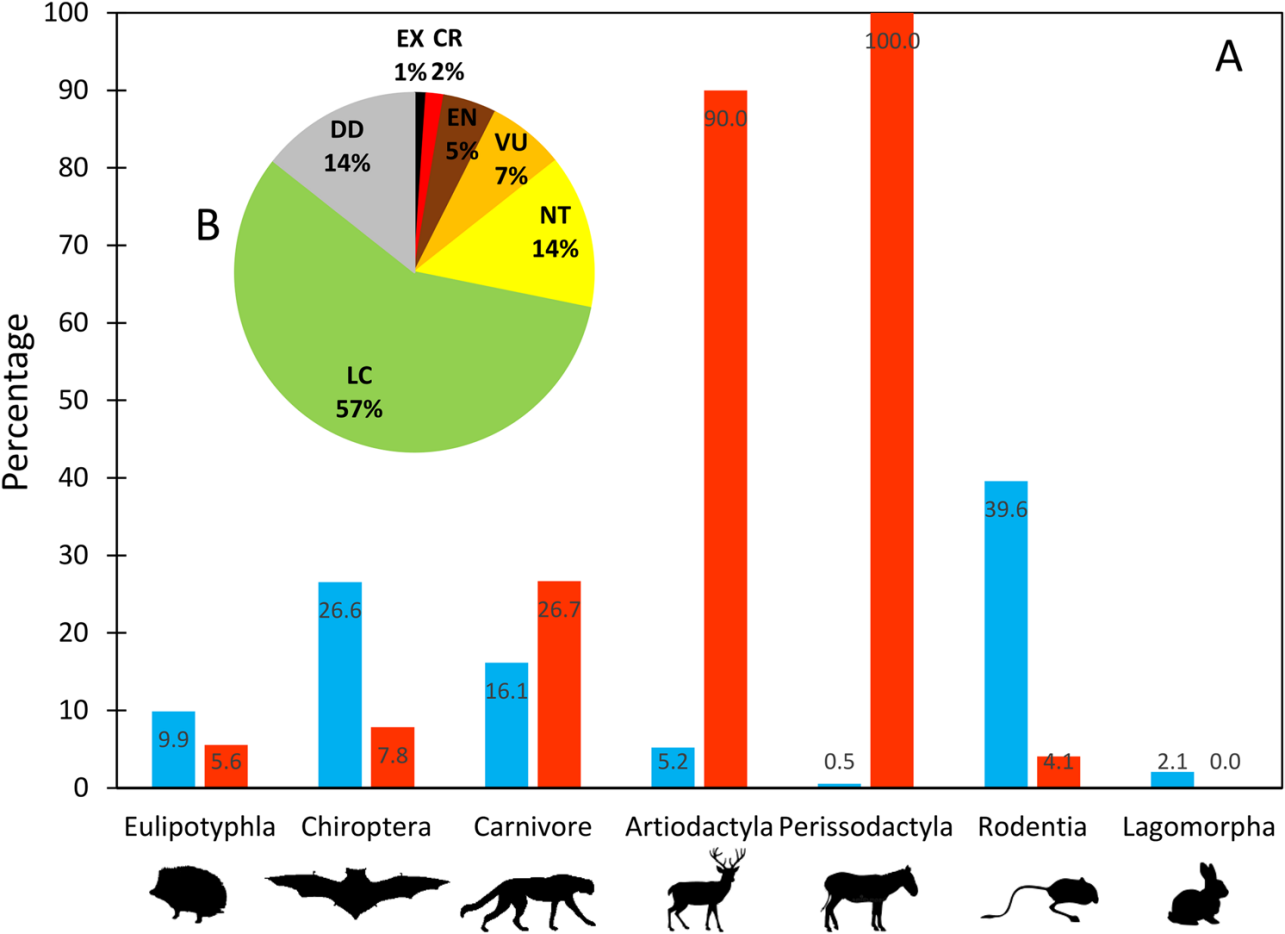
(**A**) Blue bars show the percentage of Iranian terrestrial mammal species in different orders and red bars show the percentage of orders listed as threatened at national level. (**B**) The pie chart shows the percentage frequency of species in each of the Iran Red List categories: Data Deficient (DD), Least Concern (LC), Near Threatened (NT), Vulnerable (VU), Endangered (EN), Critically Endangered (CR), and Extinct (EX).

As a measure of extinction risk, we followed previous studies (e.g.,^22^) in the use of classifications based on IUCN status categories. The IUCN status categories of the 179 species (Supplementary Table S1) were taken from a recent conservation assessment (Fig. 3)^13^. We used the national status rather than global status, because regional IUCN Red List is considered more suitable when developing regional-scale analyses, as regional status should reflect more accurately the extinction risk within a country^19^. Excluding species with unknown threat status (Data Deficient), a total of 156 species belonging to seven orders and 31 families categorised from Least Concern to Regionally Extinct as a continuous character (from 0–5 in increasing order of relative risk, following Fritz et al.^21^), were available for analyses (see details in Supplementary Table S1). Given that IUCN uses small geographical range for listing species as threatened (criterion B), the species’ range size was excluded from the analyses to avoid circularity ^54^.

### Statistical analyses

We first tested for evidence of phylogenetic non-independence in the model residuals potentially violating the assumptions in linear models^54^. Such control is necessary because closely related species may not represent independent samples^54^. We used generalized linear mixed models (GLMMs) with Penalized Quasi-Likelihood (*glmmPQL* function) in ‘mass’ R package^55^. Furthermore, we applied “*Moran’s I*” ^56^ using ‘ape’ R package ^45^ to ensure that the residuals of the phylogenetic model did not show signs of phylogenetic autocorrelation.

We applied generalized linear models (GLM) in R^44^ to estimate the effects of intrinsic/extrinsic variables (actual evapotranspiration rate, adult body mass, diet breadth, gestation length, habitat breadth, litter size, and trophic level) as well as direct (hunting/persecution) and indirect (human influence index) human factors on the extinction rates of mammalian species in Iran. We ran three different models: (1) all mammals, (2) small-bodied mammals and (3) large-bodied mammals. Initially, we scaled all variables and checked for multicollinearity using the variance inflation factor (VIF) using ‘car’ package and excluded variables if VIF > 3^57^. Next, we used Quasi-Akaike’s information Criterion corrected for small sample size (QAICc) approach to evaluate the best fitting models and to control for overdispersion^58^. For our final inferences, we applied multi-model averaging in ‘MuMIn’ R package^59^ and selected candidate models with ΔQAIC < 2. We report unconditional estimates and the 95% confidence interval (CI) values, and we considered predictors as significant if their 95% confidence intervals (CI) did not include zero. We measured the variables relative importance (RI) by QAICc-weighted standardized coefficients of the original model^58^. We used the odds ratio exp(*β*) to estimate the strength of the predicted effects on the extinction risk in our GLM models, indicating no effect when the odds ratio was around 1, negative effect when the ratio was less than 1 and positive effect when the ratio was higher than 1 (Table 2). This approach is often preferred over the traditional stepwise methods, which ignore model uncertainties, produces more robust inference especially because it maximises the number of species and for the use of complex trait combinations [58 and references therein].

We next used simulations (sensitivity analysis) to evaluate the performance of our models. We did this to determine whether extinction risk was randomly distributed among species, since the vulnerability of the species to human threats are not equally assessed across all species. Thus, we simulated 99-fold replications of the response variables discretely for each model by adding or subtracting a random number drawn from a Poisson distribution to the approximate mean of the response variable [with mean = 0.6]. We assumed that our original models performed well if the estimates and the relative importance of the predictors did not change in the simulation models^38^.

We first ran models across all species with adult body mass as a continuous explanatory variable. We then ran analyses separately for the small and medium-large (> ~ 1 kg, hereafter “large” following Ripple et al.^7^) species due to the pronounced differences in factors explaining extinction risk in these groups (e.g.,^29^). The category of small mammals (*n* = 117) included all species belonging to orders Eulipotyphla, Chiroptera, Rodentia (except *Hystrix indica*), one lagomorph (*Ochotona rufescens*), and three small carnivores (*Mustela nivalis*, *Urva edwardsii* and *Vormela peregusna*), while large mammals (*n* = 39) included all species belonging to the orders Artiodactyla, Carnivora (except the three species mentioned above), Perissodactyla, three lagomorphs (*Lepus sp.*), and one rodent (*Hystrix indica*) (Supplementary Table S1). Setting the cut-off at 1 kg is expected to be a good proxy for hunting and persecution, as species with body mass above this threshold (beginning with species such as hairs) are more susceptible to hunting and persecution.

## Supplementary Information

Supplementary Information.

## Data Availability

All relevant data are within the paper and its Supporting Information files.
