# Supplementary material for: Hunting and persecution drive mammal declines in Iran
Source: Sci Rep. 2022 Oct 22;12:17743. doi: 10.1038/s41598-022-22238-5 (PMC9588066; doi:10.1038/s41598-022-22238-5)

**SUPPLEMENTARY INFORMATION**

**Hunting and persecution drive mammal declines in Iran**

**Authors**

Gholam Hosein Yusefi¹²³^4^*, José Carlos Brito¹²^3^, Mahmood Soofi^5^, Kamran Safi^67^.

**Table S1 –** The 156 Iranian terrestrial mammals, the size categories (Size; S=Small, L=Large), the average Human Influence Index (HII; high values indicate high human influence), range size given by the extent of occurrence (EOO; km^2^) used to calculate HII, IUCN Regional Red List categories converted to a numerical scale (IUCN; 0=Least Concern, 1=Near Threatened, 2=Vulnerable, 3=Endangered, 4=Critically Endangered, and 5=Regionally Extinct), average adult body mass (ABM; gr), diet breadth (DB; number of diet categories eaten by each species), average gestation length (GL; days), habitat breadth (HB; number of habitat layers used by each species), average litter size (LS), trophic level (TL; 1=Herbivore, 2=Omnivore, and 3=Carnivore), average actual evapotranspiration rate (AET; in mm), and hunting vulnerability via IUCN Threats (HV; 1= rarely/never hunted or persecuted: hunting is not a threat for species , 2=occasionally hunted or persecuted: hunting is not main threat, and 3 = often hunted or persecuted: hunting is main threat). Diet categories include vertebrate, invertebrate, fruit, flowers/nectar/pollen, leaves/branches/bark, seeds, grass and roots/tubers. Habitat layers include above ground dwelling, aquatic, fossorial and ground dwelling.

| **Species** | **Order** | **Family** | **Size^** | **HII** | **EOO** | **IUCN¥** | **ABM#** | **DB#** | **GL#** | **HB#** | **LS#** | **TL#** | **AET#** | **HV** |
| --- | --- | --- | --- | --- | --- | --- | --- | --- | --- | --- | --- | --- | --- | --- |
| *Acinonyx jubatus* | Carnivora | Felidae | L | 8.33 | 2.42E+05 | 4 | 50577.92 | 1 | 92.24 | 1 | 3.28 | 3 | 595.06 | 2 |
| *Acomys dimidiatus* | Rodentia | Muridae | S | 16.06 | 1.91E+05 | 0 | 90 | 4 | 53 | 2 | 2.47 | 2 | 171.2 | 1 |
| *Apodemus hyrcanicus* | Rodentia | Muridae | S | 21.88 | 1.02E+05 | 0 | 20.66 | 6 | 23 | 2 | 5.72 | 1 | 519.29 | 1 |
| *Apodemus uralensis* | Rodentia | Muridae | S | 22.45 | 4.79E+04 | 0 | 18.26 | 6 | 23 | 2 | 5.72 | 1 | 389.88 | 1 |
| *Apodemus witherbyi* | Rodentia | Muridae | S | 15.64 | 1.21E+06 | 0 | 24.5 | 6 | 23 | 2 | 5.72 | 1 | 397.25 | 1 |
| *Arvicola amphibius* | Rodentia | Cricetidae | S | 19.44 | 3.61E+05 | 0 | 120 | 6 | 21.24 | 2 | 4.76 | 1 | 348.9 | 1 |
| *Asellia tridens* | Chiroptera | Hipposideridae | S | 15.63 | 7.80E+05 | 0 | 12.94 | 1 | 66.1 | 1 | 1 | 3 | 151.4 | 1 |
| *Barbastella barbastellus* | Chiroptera | Vespertilionidae | S | 18.95 | 1.43E+05 | 0 | 8.31 | 1 | 65.2 | 1 | 1.73 | 3 | 498.71 | 2 |
| *Barbastella capsica** | Chiroptera | Vespertilionidae | S | 15.99 | 2.79E+04 | 0 | 15.05 | 1 | 65.2 | 1 | 1.73 | 3 | 630.25 | 1 |
| *Calomyscus bailwardi* | Rodentia | Calomyscidae | S | 15.70 | 1.09E+06 | 0 | 21.38 | 6 | 31.5 | 2 | 4 | 1 | 292.44 | 1 |
| *Calomyscus elburzensis* | Rodentia | Calomyscidae | S | 13.57 | 5.62E+05 | 0 | 21.38 | 6 | 31.5 | 2 | 3 | 1 | 308 | 1 |
| *Calomyscus grandis* | Rodentia | Calomyscidae | S | 20.18 | 3.25E+03 | 1 | 21.38 | 6 | 31.5 | 2 | 3 | 1 | 300.6 | 1 |
| *Calomyscus hotsoni* | Rodentia | Calomyscidae | S | 12.52 | 2.20E+05 | 0 | 21.38 | 6 | 31.5 | 2 | 3 | 1 | 165.5 | 1 |
| *Calomyscus mystax* | Rodentia | Calomyscidae | S | 19.74 | 6.37E+02 | 1 | 21.38 | 6 | 31.5 | 2 | 4.99 | 1 | 300.6 | 1 |
| *Calomyscus urartensis* | Rodentia | Calomyscidae | S | 21.14 | 1.50E+03 | 0 | 21.38 | 6 | 31.5 | 2 | 3 | 1 | 436.71 | 1 |
| *Canis aureus* | Carnivora | Canidae | L | 15.44 | 1.76E+06 | 0 | 9658.7 | 6 | 61.24 | 1 | 3.74 | 2 | 438.02 | 1 |
| *Canis lupus* | Carnivora | Canidae | L | 15.52 | 1.73E+06 | 1 | 31756.51 | 1 | 63.5 | 1 | 4.98 | 3 | 313.33 | 3 |
| *Capra aegagrus* | Artiodactyla | Bovidae | L | 15.41 | 1.60E+06 | 3 | 47386.47 | 2 | 156 | 1 | 1.45 | 1 | 818.4 | 3 |
| *Capreolus capreolus* | Artiodactyla | Cervidae | L | 20.77 | 2.56E+05 | 3 | 22502.01 | 5 | 196 | 1 | 1.79 | 1 | 450.28 | 2 |
| *Caracal caracal* | Carnivora | Felidae | L | 14.27 | 1.25E+06 | 1 | 11964.38 | 1 | 71.47 | 1 | 2.3 | 3 | 455.81 | 2 |
| *Cervus elaphus* | Artiodactyla | Cervidae | L | 21.37 | 1.40E+05 | 3 | 240867.1 | 5 | 235.61 | 1 | 1.09 | 1 | 421.62 | 2 |
| *Chionomys nivalis* | Rodentia | Cricetidae | S | 15.00 | 8.74E+05 | 2 | 42.01 | 5 | 20.92 | 2 | 3.42 | 1 | 540.61 | 1 |
| *Cricetulus migratorius* | Rodentia | Cricetidae | S | 15.18 | 1.49E+06 | 0 | 34.6 | 6 | 12 | 2 | 5.2 | 1 | 279.95 | 1 |
| *Crocidura caspica* | Eulipotyphla | Soricidae | S | 32.62 | 4.00E+04 | 0 | 8.13 | 3 | 29 | 1 | 4.28 | 2 | 319.6 | 1 |
| *Crocidura gmelini* | Eulipotyphla | Soricidae | S | 17.08 | 1.61E+05 | 0 | 8.13 | 3 | 29 | 1 | 4.28 | 2 | 319.6 | 1 |
| *Crocidura leucodon* | Eulipotyphla | Soricidae | S | 19.13 | 3.54E+05 | 0 | 10.88 | 3 | 30.99 | 1 | 3.36 | 2 | 471.93 | 1 |
| *Crocidura suaveolens* | Eulipotyphla | Soricidae | S | 15.28 | 1.48E+06 | 0 | 7.35 | 4 | 27.15 | 1 | 3.49 | 2 | 319.6 | 1 |
| *Crocidura susiana* | Eulipotyphla | Soricidae | S | 33.07 | 2.52E+01 | 3 | 8.13 | 3 | 29 | 1 | 4.28 | 2 | 298 | 1 |
| *Crocidura zarudnyi* | Eulipotyphla | Soricidae | S | 13.80 | 2.67E+05 | 0 | 6.3 | 3 | 29 | 1 | 5.99 | 2 | 189.12 | 1 |
| *Dama mesopotamica* | Artiodactyla | Cervidae | L | 25.96 | 6.79E+02 | 4 | 57224.61 | 2 | 230 | 1 | 1 | 1 | 475.08 | 3 |
| *Dryomys nitedula* | Rodentia | Gliridae | S | 15.34 | 1.04E+06 | 0 | 29.5 | 4 | 24.5 | 3 | 3.24 | 2 | 382.11 | 1 |
| *Ellobius fuscocapillus* | Rodentia | Cricetidae | S | 12.19 | 4.94E+05 | 0 | 54.2 | 5 | 26 | 1 | 3.46 | 1 | 376.3 | 1 |
| *Ellobius lutescens* | Rodentia | Cricetidae | S | 19.36 | 4.24E+05 | 0 | 117.3 | 5 | 26 | 1 | 4.66 | 1 | 311.01 | 1 |
| *Ellobius talpinus* | Rodentia | Cricetidae | S | 18.40 | 8.40E+03 | 1 | 40 | 5 | 26 | 1 | 3.67 | 1 | 321.71 | 1 |
| *Eptesicus bottae* | Chiroptera | Vespertilionidae | S | 35.18 | 6.06E+01 | 1 | 15.66 | 1 | 65 | 1 | 1.5 | 3 | 291.75 | 1 |
| *Eptesicus serotinus* | Chiroptera | Vespertilionidae | S | 15.59 | 7.65E+05 | 0 | 23.09 | 1 | 65.44 | 1 | 1.5 | 3 | 557.37 | 1 |
| *Equus hemionus* | Perissodactyla | Equidae | L | 6.92 | 4.71E+04 | 3 | 235248.1 | 2 | 346 | 1 | 1 | 1 | 253.84 | 3 |
| *Erinaceus concolor* | Eulipotyphla | Erinaceidae | S | 15.55 | 1.10E+06 | 0 | 665.99 | 8 | 34.99 | 1 | 5.71 | 2 | 357.2 | 1 |
| *Felis chaus* | Carnivora | Felidae | L | 15.47 | 1.60E+06 | 0 | 7157.99 | 1 | 62.88 | 1 | 2.94 | 3 | 596.11 | 2 |
| *Felis margarita* | Carnivora | Felidae | L | 10.87 | 5.35E+05 | 0 | 2823.36 | 1 | 63.61 | 1 | 4.12 | 3 | 125.83 | 2 |
| *Felis silvestris* | Carnivora | Felidae | L | 20.47 | 1.00E+05 | 1 | 4573.08 | 1 | 65.49 | 1 | 3.59 | 3 | 510.78 | 2 |
| *Funambulus pennantii* | Rodentia | Sciuridae | S | 14.78 | 4.42E+04 | 0 | 102.49 | 3 | 41.77 | 3 | 2.95 | 1 | 631.74 | 1 |
| *Gazella bennettii* | Artiodactyla | Bovidae | L | 12.69 | 9.89E+05 | 3 | 18916.67 | 3 | 159.68 | 1 | 1.39 | 1 | 529.23 | 3 |
| *Gazella gazella* | Artiodactyla | Bovidae | L | 4.00 | 1.72E+00 | 3 | 21306.83 | 3 | 180 | 1 | 1.22 | 1 | 180.3 | 3 |
| *Gazella subgutturosa* | Artiodactyla | Bovidae | L | 15.21 | 1.18E+06 | 3 | 26981.37 | 3 | 159.68 | 1 | 1.39 | 1 | 208.39 | 3 |
| *Gerbillus aquilus* | Rodentia | Muridae | S | 13.41 | 1.89E+05 | 0 | 28.1 | 4 | 21 | 2 | 5.58 | 1 | 146.35 | 1 |
| *Gerbillus cheesmani* | Rodentia | Muridae | S | 17.82 | 2.15E+01 | 0 | 28 | 4 | 21 | 2 | 8 | 1 | 166.03 | 1 |
| *Gerbillus mesopotamiae* | Rodentia | Muridae | S | 23.09 | 2.67E+03 | 2 | 31 | 4 | 21 | 2 | 5.58 | 1 | 242.95 | 1 |
| *Gerbillus nanus* | Rodentia | Muridae | S | 13.34 | 1.12E+06 | 0 | 25.5 | 4 | 20.97 | 2 | 3.16 | 1 | 194.05 | 1 |
| *Glis glis* | Rodentia | Gliridae | S | 23.42 | 9.30E+04 | 0 | 128.09 | 6 | 28.19 | 1 | 5.17 | 1 | 498.45 | 2 |
| *Golunda ellioti* | Rodentia | Muridae | S | 18.74 | 5.09E+03 | 0 | 60.73 | 2 | 26.2 | 2 | 4.83 | 1 | 757.06 | 1 |
| *Hemiechinus auritus* | Eulipotyphla | Erinaceidae | S | 15.21 | 1.19E+06 | 0 | 322.04 | 3 | 37.18 | 2 | 3.74 | 2 | 267.04 | 1 |
| *Hyaena hyaena* | Carnivora | Hyaenidae | L | 15.00 | 1.60E+06 | 1 | 35070.51 | 6 | 90.5 | 1 | 2.44 | 2 | 346.34 | 2 |
| *Hypsugo savii** | Chiroptera | Vespertilionidae | S | 14.88 | 1.15E+06 | 0 | 6.3 | 1 | 44 | 1 | 2 | 3 | 475.46 | 1 |
| *Hystrix indica*‡ | Rodentia | Hystricidae | L | 15.33 | 1.68E+06 | 0 | 14298.6 | 6 | 112 | 2 | 1.73 | 2 | 386.4 | 1 |
| *Jaculus blanfordi* | Rodentia | Dipodidae | S | 12.90 | 9.46E+05 | 0 | 85 | 6 | 33 | 2 | 3.88 | 1 | 175.79 | 2 |
| *Jaculus loftusi** | Rodentia | Dipodidae | S | 15.18 | 4.36E+05 | 0 | 59.8 | 6 | 33.54 | 2 | 3.24 | 1 | 178.89 | 2 |
| *Lepus capensis*‡ | Lagomorpha | Leporidae | L | 14.80 | 1.52E+06 | 0 | 2047.11 | 3 | 41.99 | 1 | 2.44 | 1 | 426.9 | 2 |
| *Lepus europaeus*‡ | Lagomorpha | Leporidae | L | 20.45 | 1.26E+05 | 0 | 3816.43 | 3 | 41.99 | 2 | 2.14 | 1 | 407.18 | 1 |
| *Lepus tolai*‡ | Lagomorpha | Leporidae | L | 19.25 | 6.51E+04 | 0 | 1589 | 3 | 41.99 | 2 | 2.93 | 1 | 220.18 | 3 |
| *Lutra lutra* | Carnivora | Mustelidae | L | 18.07 | 6.61E+05 | 1 | 8868.69 | 1 | 64.27 | 2 | 2.01 | 3 | 507.32 | 2 |
| *Lynx lynx* | Carnivora | Felidae | L | 17.19 | 7.14E+05 | 1 | 19300 | 1 | 66.99 | 1 | 2.3 | 3 | 346.68 | 3 |
| *Martes foina* | Carnivora | Mustelidae | L | 15.44 | 1.49E+06 | 0 | 1675 | 1 | 29.86 | 1 | 3.78 | 3 | 443.88 | 3 |
| *Martes martes* | Carnivora | Mustelidae | L | 25.40 | 5.90E+04 | 0 | 1299.99 | 1 | 30.63 | 1 | 3.49 | 3 | 399.41 | 3 |
| *Meles canescens** | Carnivora | Mustelidae | L | 16.96 | 8.26E+05 | 0 | 11884.03 | 1 | 48.6 | 1 | 3.11 | 3 | 422.75 | 2 |
| *Mellivora capensis* | Carnivora | Mustelidae | L | 15.71 | 5.88E+05 | 0 | 8999.99 | 6 | 181.46 | 1 | 2.35 | 2 | 682.1 | 3 |
| *Meriones crassus* | Rodentia | Muridae | S | 14.31 | 1.23E+06 | 0 | 69.94 | 4 | 23.5 | 2 | 4.21 | 1 | 108.69 | 1 |
| *Meriones hurrianae* | Rodentia | Muridae | S | 14.45 | 7.33E+04 | 0 | 71.02 | 4 | 29.19 | 2 | 4.1 | 1 | 314.37 | 2 |
| *Meriones libycus* | Rodentia | Muridae | S | 15.26 | 1.63E+06 | 0 | 91.33 | 4 | 25.43 | 2 | 4.54 | 1 | 167.3 | 1 |
| *Meriones meridianus* | Rodentia | Muridae | S | 12.41 | 2.91E+05 | 0 | 52.26 | 4 | 23.95 | 2 | 5.64 | 1 | 207.58 | 1 |
| *Meriones persicus* | Rodentia | Muridae | S | 15.17 | 1.67E+06 | 0 | 90.5 | 4 | 28 | 2 | 5.83 | 1 | 263.87 | 1 |
| *Meriones tristrami* | Rodentia | Muridae | S | 19.56 | 3.65E+05 | 0 | 111.62 | 4 | 23.99 | 2 | 5.8 | 1 | 323.04 | 1 |
| *Meriones vinogradovi* | Rodentia | Muridae | S | 20.06 | 2.84E+05 | 0 | 117 | 4 | 21.89 | 2 | 7.48 | 1 | 406.51 | 1 |
| *Mesocricetus brandti* | Rodentia | Cricetidae | S | 20.52 | 1.98E+05 | 0 | 198 | 4 | 15 | 2 | 9.01 | 1 | 441.25 | 2 |
| *Microtus afghanus** | Rodentia | Cricetidae | S | 19.94 | 15707.15 | 0 | 46 | 6 | 20 | 2 | 3.4 | 1 | 266.03 | 1 |
| *Microtus irani* | Rodentia | Cricetidae | S | 15.81 | 9.60E+05 | 0 | 26.9 | 5 | 20 | 2 | 4.99 | 1 | 301 | 1 |
| *Microtus mystacinus** | Rodentia | Cricetidae | S | 20.37 | 3.77E+04 | 0 | 35.49 | 5 | 20 | 2 | 4.99 | 1 | 410.37 | 1 |
| *Microtus obscurus** | Rodentia | Cricetidae | S | 20.49 | 2.75E+05 | 0 | 26.9 | 5 | 21 | 2 | 4.99 | 1 | 415.16 | 1 |
| *Microtus paradoxus* | Rodentia | Cricetidae | S | 17.42 | 3.47E+04 | 0 | 25.8 | 5 | 20 | 2 | 4.99 | 1 | 385.2 | 1 |
| *Microtus qazvinensis* | Rodentia | Cricetidae | S | 19.50 | 6.03E+04 | 0 | 26.9 | 5 | 20 | 2 | 4.99 | 1 | 385.2 | 1 |
| *Microtus schelkovnikovi* | Rodentia | Cricetidae | S | 27.86 | 1.18E+04 | 1 | 26.9 | 5 | 20 | 2 | 4.99 | 1 | 525.79 | 2 |
| *Microtus socialis* | Rodentia | Cricetidae | S | 17.65 | 7.04E+05 | 0 | 48 | 5 | 19.5 | 2 | 5.51 | 1 | 329.61 | 1 |
| *Microtus transcaspicus* | Rodentia | Cricetidae | S | 19.50 | 2.89E+04 | 0 | 38.8 | 5 | 20 | 2 | 2.1 | 1 | 329.37 | 1 |
| *Miniopterus pallidus** | Chiroptera | Miniopteridae | S | 15.86 | 1063104 | 2 | 11.46 | 1 | 182.5 | 1 | 1 | 3 | 679.41 | 1 |
| *Mus macedonicus* | Rodentia | Muridae | S | 20.52 | 3.47E+05 | 0 | 12.5 | 1 | 19.6 | 2 | 5.54 | 1 | 392.56 | 1 |
| *Mus musculus* | Rodentia | Muridae | S | 15.29 | 1.74E+06 | 0 | 19.3 | 1 | 19.6 | 2 | 5.54 | 3 | 407 | 1 |
| *Mustela nivalis*† | Carnivora | Mustelidae | S | 17.33 | 7.82E+05 | 0 | 78.45 | 1 | 36.49 | 1 | 5.07 | 3 | 334.68 | 3 |
| *Myomimus personatus* | Rodentia | Gliridae | S | 19.46 | 7.40E+03 | 1 | 35 | 4 | 30 | 2 | 5.5 | 1 | 267.41 | 1 |
| *Myomimus setzeri* | Rodentia | Gliridae | S | 18.97 | 5.06E+04 | 0 | 35 | 4 | 30 | 2 | 5.5 | 1 | 335.74 | 1 |
| *Myotis bechsteinii* | Chiroptera | Vespertilionidae | S | 24.93 | 1.85E+03 | 1 | 9.47 | 1 | 55 | 1 | 1 | 3 | 515.69 | 2 |
| *Myotis blythii* | Chiroptera | Vespertilionidae | S | 15.23 | 1.40E+06 | 0 | 23.82 | 1 | 55 | 1 | 1 | 3 | 416.49 | 2 |
| *Myotis capaccinii* | Chiroptera | Vespertilionidae | S | 18.68 | 1.77E+05 | 0 | 8.15 | 1 | 54.99 | 1 | 1 | 3 | 431.11 | 2 |
| *Myotis davidii* | Chiroptera | Vespertilionidae | S | 19.03 | 3.70E+05 | 0 | 6 | 1 | 55 | 1 | 1 | 3 | 470.9 | 1 |
| *Myotis emarginatus* | Chiroptera | Vespertilionidae | S | 14.69 | 1.37E+06 | 0 | 7.56 | 1 | 55 | 1 | 1 | 3 | 440.36 | 2 |
| *Myotis nattereri* | Chiroptera | Vespertilionidae | S | 20.96 | 1.08E+03 | 1 | 7.25 | 1 | 55 | 1 | 1 | 3 | 461.22 | 1 |
| *Myotis schaubi* | Chiroptera | Vespertilionidae | S | 20.44 | 8.69E+04 | 0 | 12 | 1 | 55 | 1 | 1 | 3 | 560.81 | 1 |
| *Neomys milleri** | Eulipotyphla | Soricidae | S | 20.04 | 4.67E+03 | 0 | 15 | 2 | 20 | 3 | 5 | 1 | 523.97 | 1 |
| *Nesokia indica* | Rodentia | Muridae | S | 14.95 | 1.48E+06 | 0 | 126.3 | 2 | 17 | 2 | 4.18 | 1 | 269.32 | 1 |
| *Nyctalus leisleri* | Chiroptera | Vespertilionidae | S | 15.32 | 4.35E+05 | 0 | 12.47 | 1 | 72 | 1 | 1.73 | 3 | 498.35 | 1 |
| *Nyctalus noctula* | Chiroptera | Vespertilionidae | S | 18.26 | 1.89E+05 | 0 | 28.48 | 1 | 72.24 | 1 | 1.33 | 3 | 506.65 | 1 |
| *Nyctinomus aegyptiacus** | Chiroptera | Molossidae | S | 13.60 | 1.47E+04 | 0 | 17.63 | 1 | 85.52 | 1 | 1 | 3 | 606.46 | 1 |
| *Ochotona rufescens* | Lagomorpha | Ochotonidae | S | 15.07 | 1.28E+06 | 0 | 250 | 3 | 25.75 | 2 | 6.05 | 1 | 259.09 | 3 |
| *Otocolobus manul* | Carnivora | Felidae | L | 14.92 | 9.32E+05 | 0 | 3050 | 1 | 76.08 | 1 | 4.83 | 3 | 291.26 | 2 |
| *Otonycteris hemprichii* | Chiroptera | Vespertilionidae | S | 13.46 | 9.49E+05 | 0 | 21.98 | 2 | 110 | 1 | 1.38 | 3 | 199.92 | 1 |
| *Ovis vignei** | Artiodactyla | Bovidae | L | 13.46 | 1.03E+06 | 3 | 113998.7 | 2 | 165 | 1 | 1.22 | 1 | 267.71 | 3 |
| *Panthera leo* | Carnivora | Felidae | L | 19.66 | 1.08E+05 | 5 | 158623.9 | 1 | 108.74 | 1 | 2.75 | 3 | 902.1 | 3 |
| *Panthera pardus* | Carnivora | Felidae | L | 15.06 | 1.57E+06 | 3 | 52399.99 | 1 | 96.74 | 1 | 2.14 | 3 | 818.43 | 3 |
| *Panthera tigris* | Carnivora | Felidae | L | 21.70 | 1.10E+05 | 5 | 161914.7 | 1 | 105.19 | 1 | 2.6 | 3 | 1000.12 | 3 |
| *Paraechinus aethiopicus* | Eulipotyphla | Erinaceidae | S | 4.00 | 1.38E+01 | 3 | 352.4 | 2 | 36.7 | 2 | 3.49 | 3 | 151.3 | 1 |
| *Paraechinus hypomelas* | Eulipotyphla | Erinaceidae | S | 14.60 | 1.40E+06 | 0 | 213 | 3 | 36.7 | 2 | 3.74 | 2 | 250.44 | 1 |
| *Pipistrellus kuhlii* | Chiroptera | Vespertilionidae | S | 15.31 | 1.63E+06 | 0 | 6.07 | 1 | 56 | 1 | 1.21 | 3 | 354.8 | 1 |
| *Pipistrellus pipistrellus* | Chiroptera | Vespertilionidae | S | 15.88 | 1.04E+06 | 0 | 5.3 | 1 | 44 | 1 | 1.37 | 3 | 424.73 | 1 |
| *Pipistrellus pygmaeus* | Chiroptera | Vespertilionidae | S | 32.83 | 1.10E+04 | 1 | 5.3 | 1 | 44 | 1 | 1.37 | 3 | 460.98 | 1 |
| *Pygeretmus pumilio* | Rodentia | Dipodidae | S | 19.11 | 3.56E+03 | 2 | 52.24 | 2 | 27.5 | 2 | 3.35 | 1 | 248.84 | 1 |
| *Rattus norvegicus* | Rodentia | Muridae | S | 14.61 | 1.27E+06 | 0 | 282.89 | 1 | 21.74 | 2 | 8.99 | 1 | 607.3 | 1 |
| *Rattus pyctoris* | Rodentia | Muridae | S | 17.02 | 3.79E+02 | 1 | 19.9 | 1 | 22.5 | 2 | 4.23 | 1 | 607.3 | 1 |
| *Rattus rattus* | Rodentia | Muridae | S | 15.85 | 1.12E+06 | 0 | 142.68 | 5 | 23.45 | 2 | 5.88 | 2 | 607.3 | 1 |
| *Rhinolophus blasii* | Chiroptera | Rhinolophidae | S | 15.31 | 1.35E+06 | 0 | 10.29 | 1 | 91.24 | 1 | 1 | 3 | 507.11 | 2 |
| *Rhinolophus euryale* | Chiroptera | Rhinolophidae | S | 15.83 | 9.32E+05 | 0 | 9.25 | 1 | 92.54 | 1 | 1 | 3 | 493.24 | 2 |
| *Rhinolophus ferrumequinum* | Chiroptera | Rhinolophidae | S | 15.56 | 1.37E+06 | 2 | 22.59 | 1 | 91.24 | 1 | 1 | 3 | 510.66 | 1 |
| *Rhinolophus hipposideros* | Chiroptera | Rhinolophidae | S | 15.39 | 1.25E+06 | 2 | 4.57 | 1 | 75.51 | 1 | 1 | 3 | 466.96 | 1 |
| *Rhinolophus mehelyi* | Chiroptera | Rhinolophidae | S | 18.77 | 2.87E+05 | 0 | 14.03 | 1 | 87.6 | 1 | 1 | 3 | 451.06 | 1 |
| *Rhinopoma hardwickii* | Chiroptera | Rhinopomatidae | S | 17.23 | 3.69E+05 | 0 | 13.1 | 1 | 98.28 | 1 | 1 | 3 | 327 | 1 |
| *Rhinopoma microphyllum* | Chiroptera | Rhinopomatidae | S | 17.08 | 4.14E+05 | 0 | 28.02 | 1 | 114.87 | 1 | 1 | 3 | 292.65 | 1 |
| *Rhinopoma muscatellum* | Chiroptera | Rhinopomatidae | S | 15.45 | 8.46E+05 | 0 | 9.13 | 1 | 106.5 | 1 | 1 | 3 | 247.42 | 1 |
| *Rhombomys opimus* | Rodentia | Muridae | S | 14.65 | 1.20E+06 | 0 | 12.8 | 5 | 27.5 | 2 | 4.59 | 1 | 198.64 | 1 |
| *Rhyneptesicus nasutus** | Chiroptera | Vespertilionidae | S | 18.14 | 1.47E+05 | 0 | 7.91 | 1 | 65 | 1 | 1.5 | 3 | 256.39 | 1 |
| *Rousettus aegyptiacus* | Chiroptera | Pteropodidae | S | 15.81 | 2.86E+05 | 0 | 134 | 3 | 123.39 | 1 | 1.07 | 1 | 828.48 | 2 |
| *Scarturs elater** | Rodentia | Dipodidae | S | 14.86 | 1.40E+06 | 0 | 41 | 6 | 25 | 2 | 4.47 | 1 | 242.11 | 1 |
| *Scarturs euphratica** | Rodentia | Dipodidae | S | 35.60 | 2.56E+01 | 1 | 66 | 6 | 25 | 2 | 6.4 | 1 | 314 | 2 |
| *Scarturs hotsoni** | Rodentia | Dipodidae | S | 10.43 | 4.66E+05 | 0 | 75 | 6 | 25 | 2 | 5.6 | 1 | 132.7 | 1 |
| *Scarturs vinogradovi** | Rodentia | Dipodidae | S | 12.75 | 4.18E+05 | 0 | 72 | 6 | 25 | 2 | 5.6 | 1 | 252.93 | 1 |
| *Scarturs williamsi** | Rodentia | Dipodidae | S | 17.10 | 6.59E+05 | 0 | 91 | 6 | 25 | 2 | 4.9 | 1 | 235.4 | 1 |
| *Sciurus anomalus* | Rodentia | Sciuridae | S | 18.03 | 1.14E+05 | 1 | 335 | 3 | 38 | 2 | 4 | 1 | 412.74 | 2 |
| *Spermophilopsis leptodactylus* | Rodentia | Sciuridae | S | 25.18 | 2.25E+01 | 1 | 495 | 5 | 45.62 | 1 | 4.5 | 1 | 224.53 | 1 |
| *Spermophilus fulvus* | Rodentia | Sciuridae | S | 15.07 | 3.92E+05 | 0 | 778.38 | 5 | 30.41 | 2 | 5.99 | 1 | 243.24 | 1 |
| *Spermophilus xanthoprymnus* | Rodentia | Sciuridae | S | 19.67 | 1.94E+03 | 0 | 280 | 5 | 25.4 | 2 | 4.99 | 1 | 426.83 | 1 |
| *Suncus etruscus* | Eulipotyphla | Soricidae | S | 15.38 | 1.45E+06 | 0 | 2.26 | 2 | 27.5 | 2 | 4 | 3 | 588.44 | 1 |
| *Suncus murinus* | Eulipotyphla | Soricidae | S | 42.38 | 6.71E+00 | 0 | 43.76 | 2 | 30.19 | 1 | 3.1 | 3 | 942.92 | 1 |
| *Sus scrofa* | Artiodactyla | Suidae | L | 15.47 | 1.76E+06 | 0 | 84471.54 | 5 | 115.2 | 1 | 4.52 | 2 | 567.82 | 2 |
| *Tadarida teniotis* | Chiroptera | Molossidae | S | 14.82 | 1.27E+06 | 0 | 28.07 | 1 | 80 | 1 | 1 | 3 | 450.08 | 1 |
| *Talpa talyschensis** | Eulipotyphla | Talpidae | S | 19.46 | 9.07E+03 | 1 | 128 | 2 | 28 | 1 | 3.49 | 3 | 376 | 1 |
| *Taphozous nudiventris* | Chiroptera | Emballonuridae | S | 15.32 | 6.04E+05 | 0 | 32.49 | 1 | 100.11 | 1 | 1 | 3 | 658.19 | 1 |
| *Taphozous perforatus* | Chiroptera | Emballonuridae | S | 17.68 | 3.52E+04 | 0 | 24.43 | 1 | 91.24 | 1 | 1 | 3 | 801.94 | 1 |
| *Tatera indica* | Rodentia | Sciuridae | S | 14.24 | 1.36E+06 | 0 | 146.6 | 4 | 25.25 | 2 | 4.99 | 2 | 501.55 | 1 |
| *Triaenops persicus* | Chiroptera | Rhinonycteridae | S | 17.35 | 5.90E+04 | 0 | 13.18 | 1 | 91.25 | 1 | 1 | 3 | 949.46 | 1 |
| *Ursus arctos* | Carnivora | Ursidae | L | 17.14 | 7.89E+05 | 3 | 196287.5 | 6 | 227.56 | 1 | 2.24 | 2 | 292.87 | 2 |
| *Ursus thibetanus* | Carnivora | Ursidae | L | 13.90 | 1.49E+05 | 4 | 99714.19 | 3 | 199.92 | 1 | 1.5 | 1 | 861.13 | 2 |
| *Urva auropanctata**† | Carnivora | Herpestidae | S | 13.64 | 4.82E+05 | 0 | 750 | 6 | 48.48 | 1 | 2.21 | 2 | 728.84 | 1 |
| *Urva edwardsii** | Carnivora | Herpestidae | L | 15.84 | 1.13E+06 | 0 | 1304.67 | 6 | 63.09 | 1 | 2 | 2 | 675.31 | 1 |
| *Vespertilio murinus* | Chiroptera | Vespertilionidae | S | 18.17 | 2.01E+05 | 0 | 15.42 | 1 | 52.14 | 1 | 1.5 | 3 | 367.71 | 1 |
| *Vormela peregusna*† | Carnivora | Mustelidae | S | 14.87 | 9.98E+05 | 0 | 594.13 | 1 | 60.83 | 1 | 5.07 | 3 | 299.26 | 2 |
| *Vulpes cana* | Carnivora | Canidae | L | 13.65 | 9.24E+05 | 1 | 988.5 | 6 | 57.35 | 1 | 2 | 2 | 240.12 | 2 |
| *Vulpes corsac* | Carnivora | Canidae | L | 18.48 | 1.10E+03 | 2 | 2615.33 | 6 | 54.99 | 1 | 5.62 | 2 | 230.03 | 3 |
| *Vulpes rueppellii* | Carnivora | Canidae | L | 13.13 | 8.31E+05 | 0 | 3249.97 | 1 | 52 | 1 | 2.12 | 3 | 131.57 | 3 |
| *Vulpes vulpes* | Carnivora | Canidae | L | 15.40 | 1.77E+06 | 0 | 4820.36 | 1 | 52.5 | 1 | 4.59 | 3 | 407.98 | 1 |

*Species listed under different name inside the phylogeny of mammals provided by Faurby and Svening (2015). These species had changes in classification or nomenclature (See Yusefi et al. 2019 for details).

#Variables collected in PanTHERIA database. For details in variables definition see PanTHERIA database (Jones et al. 2009).

¥ IUCN Regional Red List information gathered from Yusefi et al. (2019).

^We used the median body weight of 1 kg to distinguish between small- and large-sized species (following Fisher et al., 2014). The small mammals (*n* = 117 species) included all species belonging to orders Eulipotyphla, Chiroptera, Rodentia (except *Hystrix indica*) and one lagomorph (*Ochotona rufescens*), plus three carnivores (*Urva edwardsii*, *Mustela nivalis* and *Vormela peregusna*), and large mammals (*n* = 39) included all species belonging to Carnivora, Perissodactyla and Artiodactyla, plus three lagomorphs (*Lepus capensis*, *L. europaeus* and *L. tolai*) and one rodent (*Hystrix indica*).

†Carnivora species listed as small mammals.

‡Lagomorph and rodent species listed as large mammals.

**References:**

Faurby S, Svenning J-C A species-level phylogeny of all extant and late Quaternary extinct mammals using a novel heuristic-hierarchical Bayesian approach. Mol. Phylogenet. Evol. **84**:14–26 (2015).

Fisher DO, et al. The current decline of tropical marsupials in Australia: is history repeating? Glob. Eco. Biogeogr. **23**:181–90 (2014)

Jones KE, et al. PanTHERIA: a species-level data-base of life history, ecology, and geography of extant and recently extinct mammals. Ecology **90**: 2648–2648 (2009).

Yusefi GH, Faizolahi K, Darvish J, Safi K, Brito JC (2019) The species diversity, distribution and conservation status of the terrestrial mammals of Iran. J Mammal 100:55–71.

**Figure S1 –** Distribution of all occurrence records of terrestrial mammals in Iran, elevational variation, and geographic location of Iran within global context (small inset).


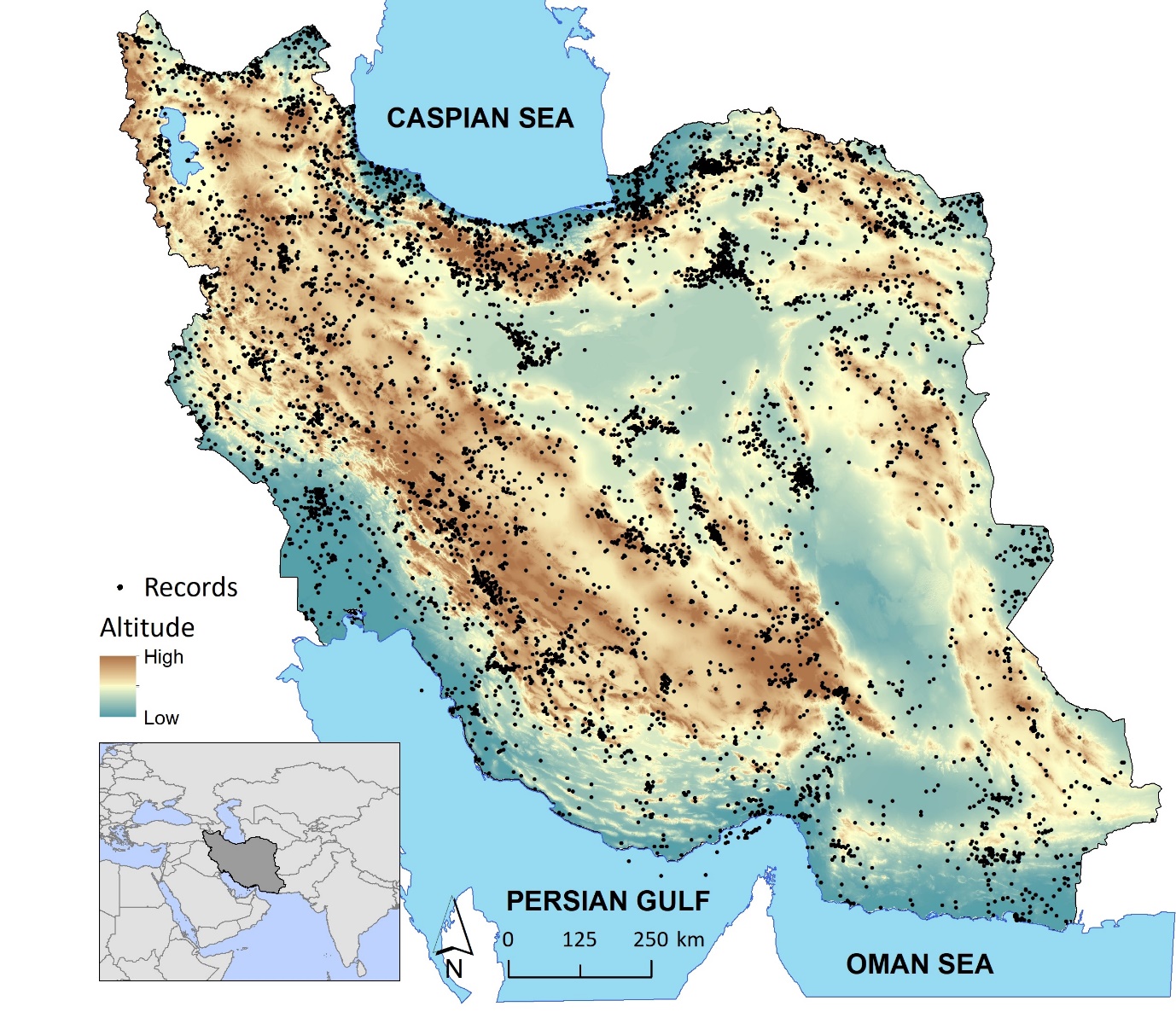


**Figure S2 –** Human influence index (HII). HII was modelled by aggregating information on several human presence and activity proxies, including population density, land transformation, accessibility.


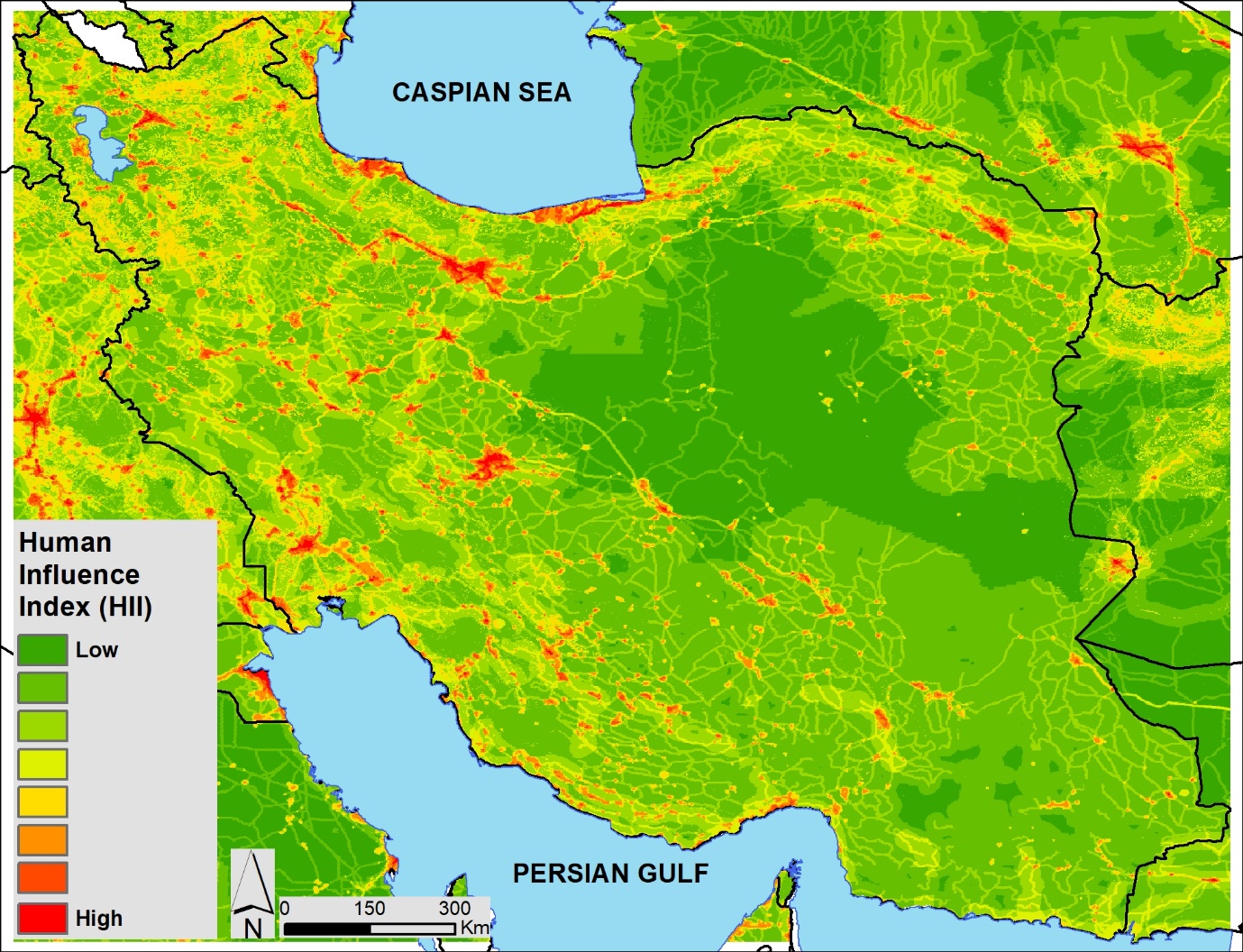

Supplement: Supplementary file 1 — Supplementary Information. [file 41598_2022_22238_MOESM1_ESM.docx]
